# Smoking *p66Shc* Knocked Out Mice Develop Respiratory Bronchiolitis with Fibrosis but Not Emphysema

**DOI:** 10.1371/journal.pone.0119797

**Published:** 2015-03-19

**Authors:** Benedetta Lunghi, Giovanna De Cunto, Eleonora Cavarra, Silvia Fineschi, Barbara Bartalesi, Giuseppe Lungarella, Monica Lucattelli

**Affiliations:** Department of Life Sciences, Section of Experimental Pathology, University of Siena, Siena, Italy; Institute of Lung Biology and Disease (iLBD), Helmholtz Zentrum München, GERMANY

## Abstract

The adaptor protein p66Shc regulates intracellular oxidant levels through the modulation of a forkhead-related transcription factor (FOXO3a). The genetic ablation of *p66^Shc^* (*p66^Shc–/–^*) renders mice resistant to oxidative stress and p53-dependent apoptosis. We investigated whether *p66^Shc^* ablation in mice modifies lung cellular and molecular responses to cigarette smoke (CS) exposure. No differences between wild type (WT) and *p66^Shc–/–^* mice were observed in terms of inflammation and oxidant burden after acute CS exposure; however,*p66^Shc^* ablation modifies specific features of chronic inflammation induced by repeated exposure to CS. Unlike WT mice, *p66^Shc–/–^* mice did not develop emphysema, showing protection toward oxidative damage to DNA and apoptosis as revealed by a trivial 8-hydroxyguanosine staining and faint TUNEL and caspase-3 positivity on alveolar epithelial cells. Unexpectedly, CS exposure in *p66^Shc–/–^* mice resulted in respiratory bronchiolitis with fibrosis in surrounded alveoli. Respiratory bronchiolitis was characterized by peribronchiolar infiltrates of lymphocytes and histiocytes, accumulation of ageing pigmented macrophages within and around bronchioles, and peribronchiolar fibrosis. The blockage of apoptosis interferes with the macrophage “clearance” from alveolar spaces, favouring the accumulation of aging macrophages into alveoli and the progressive accumulation of iron pigment in long-lived senescent cells. The presence of areas of interstitial and alveolar fibrosis in peripheral parenchyma often accompanied the bronchiolar changes. Macrophages from smoking *p66^Shc–/–^* mice elaborate M2 cytokines (i.e., IL-4 and IL-13) and enzymes (i.e., chitinase and arginase I), which can promote TGF-beta expression, collagen deposition, and fibrosis in the surrounding areas. We demonstrate here that resistance to oxidative stress and p53-dependent apoptosis can modify tissue responses to CS caused by chronic inflammation without influencing early inflammatory response to CS exposure.

## Introduction

Cigarette smoke (CS) is considered to be the main causative factor of chronic obstructive pulmonary disease (COPD) in man [1, 2, 3]. CS induces inflammation and DNA damage [4, 5], resulting in cell growth inhibition, apoptosis and cellular senescence [6]. Reactive oxygen species (ROS) (i.e. superoxide anions, hydroxyl radicals and H_2_O_2_) and cytotoxic by-products of oxidation reactions [including acrolein and hydroperoxides] [2, 3] are the most recognized mediators of lung cell damage under smoking conditions. In support of the free radical theory of emphysema, antioxidant treatment or over-expression of antioxidant genes protects from smoke-induced lung emphysema in mice [7–9].

Accumulating evidence suggests that the adaptor protein p66Shc, recently involved in lung development [10], regulates intracellular oxidant levels in mammalian cells through the regulation of a forkhead related transcription factor (FKHRL1, also called FOXO3a)[11–15]. Recent studies suggest that FOXO3a is involved in the transactivation of a number of antioxidant enzymes and stress-related gene products [12] and that FOXO3 deficiency leads to increased susceptibility to CS resulting in development of COPD [16]. Some effects of ROS are mediated by the FOXO transcription factors, which are known to control cell cycle, cell death, and stress detoxification by regulating transcription of different sets of genes [13]. In particular, H_2_O_2_ has been reported to down-regulate genes involved in both H_2_O_2_ scavenging and oxidative stress resistance (e.g., catalase and MnSOD) by inactivating FOXO3a through a p66Shc–dependent phosphorylation [12, 14]. Thus, oxidant-mediated FOXO3a inhibition requires p66Shc and the expression of p66Shc correlates with the intensity of oxidative damage in several organs such as lung, liver, skin, etc [14]. The ablation of *p66* ^*Shc*^ from mouse genome by gene targeting (*p66* ^*Shc−/−*^) renders mice resistant to oxidative stress in vivo upon treatment with paraquat [12, 15] and amazingly increases 70^th^ percentile survival in respect to other strains without influencing the life span [17]. Consistently, *p66* ^*Shc−/−*^ mice show reduced levels of systemic (isoprostane) and tissue (nitrotyrosines, 8-oxo-dG) oxidative stress markers [12, 14, 18, 19] and enhanced resistance to oxidative stress induced by carbon tetrachloride [20], ethanol [21], hypercholesterolemia (atherogenesis) [18], acute ischemia [22], and angiotensin II [23]. It is also observed that *p66* ^*Shc−/−*^ cells have enhanced resistance to apoptosis since p66Shc acts as a downstream target of the tumour suppressor p53 and regulates p53-dependent apoptosis [15].

The aim of our study was to investigate whether the enhanced resistance to apoptosis and to oxidative stress in *p66* ^*Shc−/−*^ mice modifies the cellular and molecular responses to CS and confers protection against the pulmonary changes induced by CS exposure. This was done by comparing the effects of acute and chronic exposure to CS in wild type (WT) as well as *p66* ^*Shc−/−*^ mice.

## Materials and Methods

### Ethics statement

Animal experiments were conducted in conformity with the “Guiding Principles for Research Involving Animals and Human Beings”. The Ethics Committee of the University of Siena approved the protocol.

### Mice

Dr. P.G. Pellicci at the European Institute of Oncology, Milan, kindly provided 129 *p66* ^*Shc*^ knockout breeding pairs for this project, and a colony of these mice is maintained in our animal facility. The breading stocks were backcrossed onto 129/SVEv mice (supplied by Charles River Italia, Calco, Italy) to full congenic status. Heterozygous Shc KO mice were mated to produce the homozygous Shc KO (*p66* ^*Shc−/−*^), and WT mice used for the study. The animals were bred at the University of Siena, Siena, Italy. The mice were housed in a light (07:00–19:00) and temperature controlled (18°–22°C) environment, and food (Mucedola Global Diet 2018, Harlan, Italy) and water were provided for consumption *ad libitum*.

### Determination of basal levels of Superoxide Dismutase (SOD), Catalase and Glutathione Peroxidase (GPx) activities as well as p66 Shc expression in mouse lungs

The basal values of SOD, Catalase and GPx activities in lung samples from 6 WT and 6 *p66* ^*Shc−/−*^ mice were determined colorimetrically by using commercially available kits according to manifactures’ protocols. After sacrifice, lung were perfused with PBS to remove erythrocytes, excised, homogenized in ice using Polytron homogenizer (Kinematica, Littau, Switzerlad), and centrifuged at 10,000g for 30min. The supernatant was removed and analysed for enzyme activities. SOD activity assayed by using Bioxytech SOD-525 (Oxis International Inc, Beverly Hills, CA, USA). Catalase activity and glutathione peroxidase were analysed by using CAT Assay (#700910) and GPx Assay (#703102) kits from Cayman Chemical (Ann Arbor, Michigan, USA).

At the time of sacrifice, samples of lungs were taken from WT and *p66* ^*Shc−/−*^ mice in order to evaluate by Western blotting the expression of p66 Shc protein in WT and genetically modified animals. In particular, tissues were homogenized in a 75 mM sucrose, 225 mM mannitol, 0.1% Triton X100, 30 mM Tris [pH 7.4] buffer containing inhibitors of proteases (1 mM PMSF and protein protease inhibitor cocktail) and phosphatases (1 mM Na3VO4, 10 mM NaF). After that, equal portion of pre-chilled lysis buffer (50 mM Tris, pH 7.5, 150 mM NaCl, 1% Triton, 0.1% SDS, 1% sodium deoxycholate) containing proteases and phosphatases inhibitors was added to each homogenate. Samples were centrifuged at 14,000g for 20 min at 4°C to remove debris. Protein concentration in tissue lysates was determined using Bradford method. Samples for SDS–PAGE were denaturated in a reducing Laemmli loading buffer at 95°C for 5 min. Tissue lysates were electrophoretically separated in 8% or 10% SDS–polyacrylamide gel (BioRad) (25–50 μg of total protein per lane) and transferred onto PVDF membrane (BioRad). Membranes were blocked using 2–4% non-fat milk in TBS buffer with 0.01% Tween-20 (Sigma–Aldrich) for 1 h. Proteins were detected with anti-p66Shc rabbit polyclonal antibodies (1:1000, EDM Millipore Corporation, Temecula, CA, USA) followed by secondary anti-mouse or anti-rabbit IgG conjugated to horseradish peroxidase (1:10000) [Roche Molecular Biochemicals, Milan, Italy]. The blot was developed using the enhanced chemiluminescence method (ECL-Kit Lumi-LightPlus, Roche Molecular Biochemicals, Milan, Italy) according to the manufacturer’s instructions.

### Acute Exposure to Cigarette Smoke

In the acute study, 8 mice for each experimental group were exposed to the smoke of 5 cigarettes/day in 20 minutes for three days in especially designed cages, according to Cardini et al. [24]. Control groups were exposed to room air in the same experimental conditions. Immediately after last treatments, blood samples were drawn from the right ventricle of the animals under light ether anesthesia. The trachea was isolated and then cannulated with a 20-gauge blunt needle. With the aid of a peristaltic pump (P-1 Pharmacia), the lungs were lavaged in situ three times with 0.6 ml of saline solution. The average fluid recovery was 95%. Macrophage and neutrophil cell counts were performed with a hemocytometer on bronchoalveolar lavage fluids (BALFs). Cell-free BALF obtained after centrifugation at 600 g for 15 min was used for biochemical assays of total thiols [25], total and oxidized glutathione [26] and for determination of “Trolox equivalent antioxidant capacity” (TEAC) according to Miller et al. [27]. In particular, total and oxidized (GSSG) glutathione were assayed in cell-free BALF with the enzyme recycling method essentially as described by Tietze [25] with the following modifications. The thiol-scavenging reagent 1-methyl-2-vinylpyridinium trifluoromethanesulfonate was used instead of N-ethylmaleimide (BIOXYTECH GSH/GSSG-412, OXIS Health Products, Portland, OR). Aliquots of free-cell BALFs were analysed for keratinocyte-derived chemokine (KC, also called CXCL1 or mouse GRO-alpha homolog) by using ELISA Duo-sets from R&D System Europe Ltd.

### Chronic Exposure to Cigarette Smoke

Mice from each experimental group were exposed to the smoke of three cigarettes/day, 5 days/week for 3, 5, or 7 months (Virginia filter cigarettes: 12 mg of tar and 0.9 mg of nicotine) in especially designed cages, according to Cavarra et al. [28]. Control mice from each experimental group were exposed to air under the same conditions (sham-exposed).

### Morphology, Morphometry, BAL Cell Counts and Lung Collagen Determination

At various time intervals during the chronic exposure, 8 animals of each group were sacrificed and the lungs fixed intratracheally with formalin (5%) at a pressure of 20 cm H_2_O. Post-fixation lung volume (V) was measured by water displacement. The lungs of all animals were then processed for histological, morphometrical and immunohistochemical analysis. Lung slices were stained with hematoxylin-eosin, Masson’s trichrome and Perls’ Prussian blue reaction for ferric iron. The extent of lung injury was assessed by morphometric determination of the average interalveolar distance (mean linear intercept: Lm) [29] and of the internal surface area (ISA) [30, 31] calculated on the basis of Lm and of the post-fixation lung volume (VL), according to the formula: ISA = 4xVL/Lm [31]. For the determination of the Lm for each pair of lungs, 40 histological fields were evaluated both vertically and horizontally [32, 33]. The value of Lm is calculated as described by Campbell and Tomkieff [30] as follows: Lm = nx L/Σi, where n is the number of lines counted, L is the length of the line and Σi is the number of interalveolar intersections.

The alveolar destructive index (DI) was also determined, as described by Eidelman and coworkers [34]. DI represents for each pair of lungs the percentage of air spaces in which two or more breaks in the alveolar walls were detected. For the assessment of the DI for each pair of lungs 20 histological fields were evaluated using a standardized plane with 20 points for a total of 400 points per section [32, 33]. For the morphometric evaluations, tissue slides were analysed independently by three different observers. The slides were coded to prevent bias. Additional 5 animals for each groups were used for BAL cell counts and for total lung collagen and elastin determination assessed as hydroxyproline and desmosine, respectively, according to a method previously described in detail [35].

### Immunohistochemistry

Tissue sections (5 μm thick) were stained for S-100, fascin, TGF-beta, IL-4, IL-13, inducible nitric oxide synthase (iNOS), chitinase (ECF-L), arginase I, CD4, 8-Hydroxyguanosine (8-HG) and p16INK4a (p16) using the immunoperoxidase method. Active Caspase-3 and MAC-3 were stained using the streptavidin-alkaline phosphatase method and the streptavidin-HRP method, respectively. The primary antibodies (Ab) used were: rat monoclonal Ab to mouse Mac-3 (1:20, BD Pharmingen, Buccinasco, Italy); rat monoclonal Ab to mouse CD4 (1:1000, Abcam Ltd, Cambridge, UK); rat monoclonal Ab to mouse IL-4 (1:400, Endogen, Woburn, MA, USA); rabbit polyclonal Ab to mouse S-100 (1:200, Abcam Ltd, Cambridge, UK); rabbit polyclonal Ab to mouse TGF-beta (1:20, Insight Biotechnology LTD, Wembley, UK); rabbit polyclonal Ab to mouse iNOS (1:100, Abcam Ltd, Cambridge, UK); rabbit anti-mouse/human cleaved (active) caspase-3 (1:400, Cell Signaling Technology, Denver, MA); goat polyclonal Ab to mouse IL-13 (1:20, R&D Systems Europe, LTD, Abingdon, UK); goat polyclonal Ab to mouse ECF-L (1:250, R&D Systems Europe, LTD, Abingdon, UK); mouse monoclonal Ab to fascin (1:200, Abcam Ltd, Cambridge, UK), mouse monoclonal Ab to p16 (1:200, Abcam Ltd, Cambridge, UK); mouse monoclonal Ab to 8-HG (1:1000, Abcam Ltd, Cambridge, UK); mouse monoclonal Ab to arginase I (1:100, BD Pharmingen, Buccinasco, Italy); mouse monoclonal Ab to ceramide (1:10, Sigma Aldrich, Saint Louis, MO, USA). All sections were rinsed and incubated for 30 min at room temperature with biotinylated mouse anti-rat IgG diluted 1:100 (Abcam, Cambridge, UK) to detect Mac-3, IL-4 and CD4; with sheep anti-rabbit IgG diluted 1:200 (Sigma) to detect the expression of TGF-beta, iNOS, active Caspase-3 or S-100.

To localize mouse primary monoclonal antibodies to fascin, p16, 8-HG, arginase I and ceramide on mouse tissues we used the Vector M.O.M. immunodetection kit (Vector Laboratories, Burlinghame, CA, USA) containing a novel blocking agent designed specifically to reduce the undesired background staining. Immunostaining was revealed by using the M.O.M. detection kit with 3,3’-diaminobenzidine tetra hydrochloride (DAB) substrate. As negative controls for the immunostaining, the primary Ab was replaced by non-immunized serum.

Evaluation DNA fragmentation was performed with TUNEL assay using the *In Situ* Cell Death Detection kit (Roche Applied Science) as described by manufacturer’s manual. TUNEL-positive cells were observed under fluorescent microscopy.

The number of 8-HG and caspase 3 positive cells in lung sections (10 random microscopic fields per lung section in 5 different sections) was counted manually in a blinded manner at x 200 magnification and averaged.

### Transmission Electron Microscopy (TEM) examination of pulmonary macrophages from lung of p66 Shc KO mice

For TEM investigation, lung samples from lungs of p66 Shc KO mice after 7 months of CS were fixed in 2% glutaraldehyde in 0.1 M sodium cacodylate buffer, pH 7.2 for 2 h at 4°C, post fixed in 1% buffered osmium tetraoxide, dehydrated through a graded series of ethanol, cleared in propylene-oxide and embedded in epoxy resin [Araldite]. Semithin sections (0.5–1 μm thick) were cut on a LKB V Ultratome and stained with 1% toluidine blue. Ultrathin (~600 Å) sections from selected areas cut with a diamond knife using the same ultramicrotome, retrieved onto copper grids, double-stained with uranyl acetate and lead citrate and examined at 100 kV with a Philips 208 S transmission electron microscope.

### RNA isolation and cDNA synthesis

Total RNA was extracted from lungs of mice at 5 and 7 months after chronic exposure to room air or cigarette smoke, using TRi Reagent (Ambion, Austin, TX, USA) according to the manufacturer's instructions. Six mice for each group were used for RNA isolation. RNA was re-suspended in RT-PCR Grade Water (Ambion, Austin, Texas, USA) and the amount and purity of RNA was quantified spectrophotometrically by measuring the optical density at 260 and 280 nm. Integrity was checked by agarose gel electrophoresis.

Two micrograms of total RNA was treated with TURBO DNAse (TURBO DNA-free kit, Ambion, Austin, TX, USA) for 30 min and reverse transcribed using the RETROscript kit (Ambion, Austin, TX, USA) according to the manufacturer's instructions. Two hundredths of the final volume of reverse transcription was used for real-time RT-PCR.

### Real-time RT-PCR

Real-time RT-PCR was performed in triplicate for each sample on the MJ Opticon Monitor 2 (MJ Research Co., Waltham, MA, USA) with specific locked nucleic acid (LNA) probes from the Mouse Universal Probe Library Set (UPL probes, Roche, Indianapolis, IN, USA).

Primers were designed by using the free online ProbeFinder software (available at the Universal ProbeLibrary Assay Design Center: www.universalprobelibrary.com) that shows a pair of specific primers for each probe from the Universal ProbeLibrary set (Table 1). PCR reactions were performed in a volume of 25 l and contained 12,5 l of FastStart TaqMan Probe Master (Roche), 300nM forward and reverse primers (TIBMolbiol, Genova, Italy), 200nM UPL probes and 5 l of cDNA. Reactions were incubated at 95°C for 10 min and then amplified for 40 cycles, each cycle comprised of an incubation step at 94°C for 15s followed by 60°C for 1 min. The real-time RT-PCR assay included a no-template control and a standard curve of four serial dilution points (in steps of 10-fold) of each of the test cDNAs. The analysis of the results was based on the comparative Ct method (Ct) in which Ct represents the cycle number at which the fluorescent signal, associated with an exponential increase in PCR products, crosses a given threshold. The average of the target gene was normalized to *18S rRNA* as the endogenous housekeeping gene [36]. The combination of primers and probes provides specific amplification and detection of the target sequence in the sample (Table 1).

**Table 1:** Primers and Probes used for Real-time RT-PCR.

Gene	Primers	Amplicon’s length	UPL probe
Tgfb1	Fw 5’ TGG AGC AAC ATG TGG AAC TC-3’Rev 5’ GTC AGC AGC CGG TTA CCA-3’	73 nt	#72
IL-4	Fw 5’ CAT CGG CAT TTT GAA CGA G 3’Rev 5’CGA GCT CAC TCT CTG TGG GTG 3’	104 nt	#2
IL-13	Fw 5’ CCT CTG ACC CTT AAG GAG CTTAT3’Rev 5’ CGT TGC ACA GGG GAG TCT 3’	70 nt	#17
Chitinase-like3	Fw 5’AAG AAC ACT GAG CTA AAA ACT CTC CT 3’Rev 5’ GAG ACC ATG GCA CTG AAC G 3’	77 nt	#88
Arginase I	Fw 5’ GAA TCT GCA TGG GCA ACC 3’Rev 5’ GAA TCC TGG TAC ATC TGG GAA C 3’	73 nt	#2
Nos2	Fw 5’ CTT TGC CAC GGA CGA GAC 3’Rev 5’ TCA TTG TAC TCT GAG GGC TGA C 3’	66 nt	#13
Catalase	Fw 5’ CCT TCA AGT TGG TTA ATG CAG A 3’Rev 5’ CAA GTT TTT GAT GCC CTG GT 3’	80 nt	#34
Superoxide dismutase 2	Fw 5’ TGC TCT AAT CAG GAC CCA TTG 3’Rev 5’ GTA GTA AGC GTG CTC CCA CAC 3’	81 nt	#3
MMP-12	Fw 5’ TTG TGG ATA AAC ACT ACT GGA GGT 3’Rev5’ AAA TCA GCT TGG GGT AAG CA 3’	72 nt	#51
MMP-9	Fw 5’ TCT GAG ACA GGG TTA CCA AGG 3’Rev 5’ AAG AGC CCA GGG ATT TGC 3’	61 nt	#64
MMP-1	Fw 5’ TGT GTT TCA CAA CGG AGA CC 3’Rev 5’ GCC CAA GTT GTA GTA GTT TTC CA 3’	73 nt	#94

### Statistical Analysis

The significance of the differences was calculated using one-way analysis of variance. A p value of less than 0.05 was considered significant.

## Results

### Basal levels of Glutathione Peroxidase (GPx), superoxide dismutase (SOD) and catalase activities in lung homogenates from WT and *p66* ^*Shc−/−*^ mice

**Fig 1:**
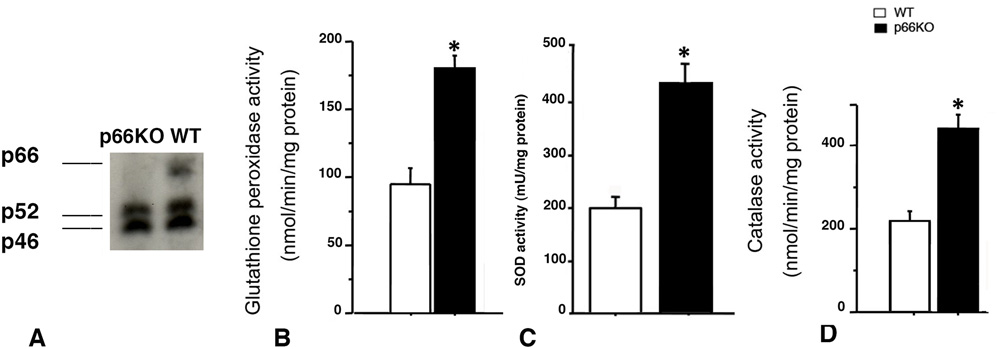
Immunoblot of p66, p52 and p46 Shc proteins and basal levels of Glutathione Peroxidase (GPx), Superoxide Dismutase (SOD) and Catalase activities in lung homogenates from WT and *p66* ^*Shc−/−*^ mice. Representative Western blot of Shc proteins in lung homogenates of WT and *p66* ^*Shc−/−*^ mice is shown in Fig. 1A. As you can see, *p66* ^*Shc−/−*^ mice exhibited significant higher levels of lung GPx, SOD and catalase activities as compared with WT mice (Fig. 1B-D).

A representative immunoblot of p66 Shc protein in lungs of WT and *p66* ^*Shc−/−*^ mice is shown in Fig. 1A. As expected, *p66* ^*Shc−/−*^ mice exhibited significant higher levels of lung GPx, SOD and catalase activities as compared with WT mice (Fig. 1B-D).

### No differences were found in BAL cell profile and inflammatory mediators between WT and *p66* ^*Shc−/−*^ mice in response to acute CS exposure

**Fig 2:**
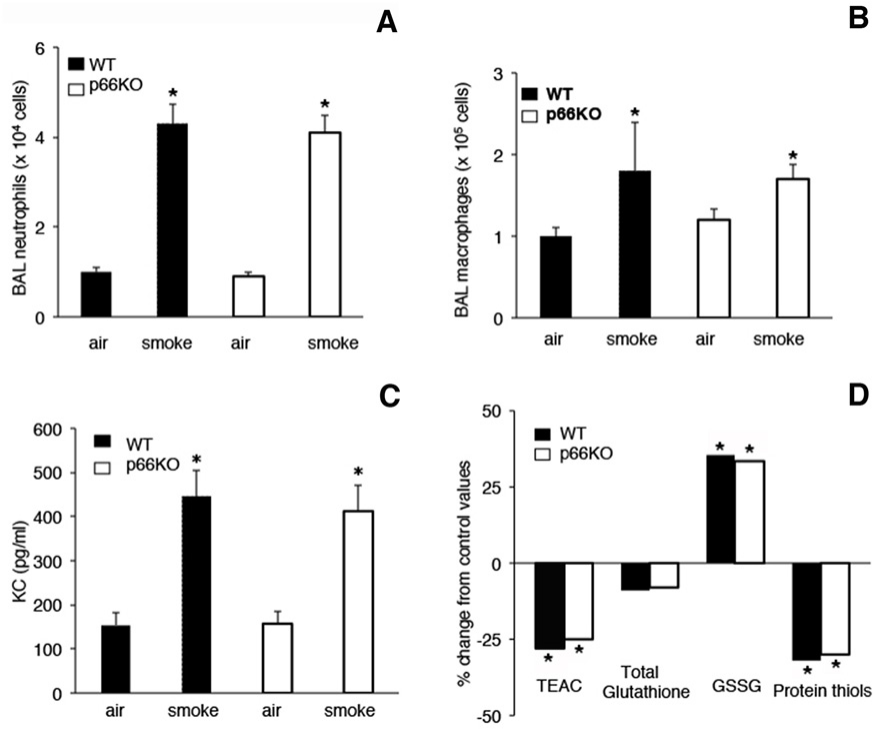
Cell counts, KC and Antioxidant Status in BALFs after Acute exposure to CS. Neutrophil (A) and Macrophage (B) counts, KC levels (C) and antioxidant status (D) in bronchoalveolar lavage (BAL) fluids of mice immediately sacrificed after smoking. Animals were exposed once a day to either smoke from five cigarettes or room air for three consecutive days. Values reported in (A-C) are means ± SD. Results in (D) are means expressed as percent of values of the Trolox equivalent antioxidant capacity (TEAC), total and oxidized glutathione (GSSG), and protein thiols found in air-exposed mice. The following values were detected in air-control WT and *p66* ^*Shc−/−*^ mice, respectively: TEAC 0.72 0.06 and 0.69 0.5 μmol/ml; total glutathione 17.49 3.34 and 15.55 3.01 nmol/mg protein; GSSG 0.51 0.03 and 0.48 0.04 nmol/mg protein; protein thiols 4.8 0.4 and 4.5 0.6 nmol/mg protein. *p< 0.05 *vs* air controls; n = 8 animal/group.

To determine the inflammatory response to acute exposure to CS in the lungs of the different groups of mice, we assessed the number of neutrophils, macrophages, and lymphocytes in BALFs by using Diff-Quick staining. As shown in Fig. 2 the number of neutrophils (A) and macrophages (B) was significantly increased in the BALFs of WT and *p66* ^*Shc−/−*^ mice exposed to CS in respect to air-controls, whereas no differences in cell counts were observed between WT and *p66* ^*Shc−/−*^ mice within control and smoking groups. Of interest, a consistent elevation of KC (the mouse homolog of human IL-8) was seen after acute CS exposure (Fig. 2C). Additionally, a significant decrease of total antioxidant capacity (TEAC) and protein thiols, as well as an increase of oxidized glutathione (GSSG) was detected after CS exposure (Fig. 2D).

No significant differences were found between the experimental groups of WT and *p66* ^*Shc−/−*^ mice exposed to air, or CS, respectively. The biochemical and cell profile we observed in BALFs of the different strains of mice after smoking is consistent with an inflammatory lung microenvironment.

### No emphysematous changes were observed in *p66* ^*Shc−/−*^ mice chronically exposed to CS

Although no substantial different responses were observed between the two strains of mice after acute challenge with CS, consistent differences were observed in lung morphology after chronic exposure to CS.

**Fig 3:**
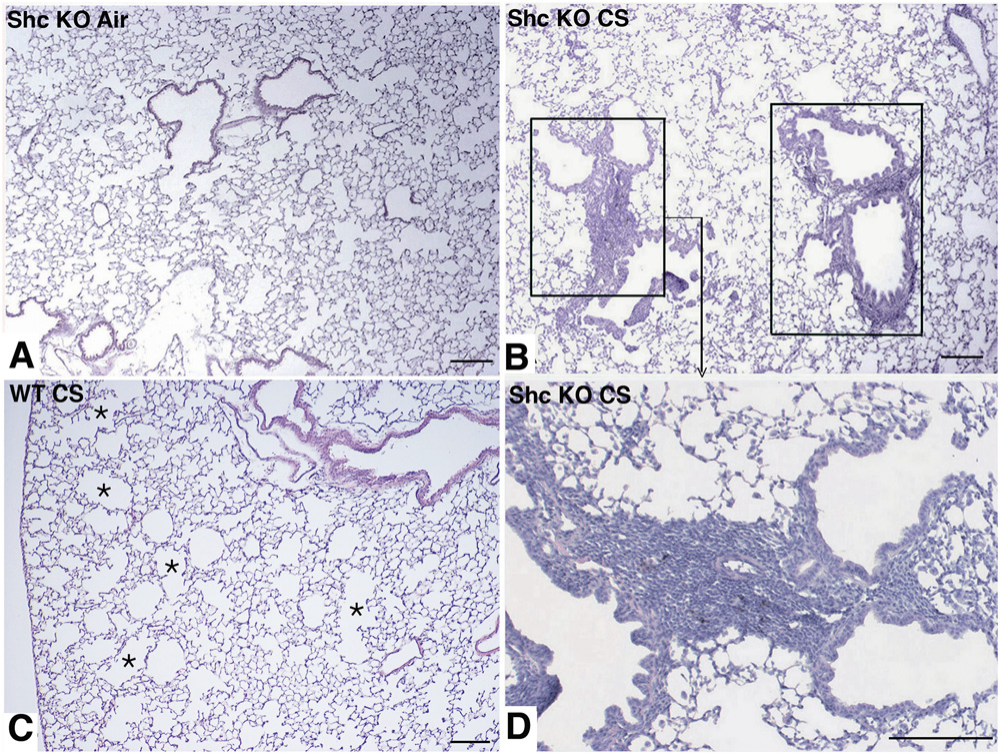
Representative lung micrographs of *p66* ^*Shc−/−*^ and WT mice at 7 months after CS or air exposure. (A) Representative lung parenchyma from an air-control *p66* ^*Shc−/−*^ mouse showing a normal architecture. (B) Smoking *p66* ^*Shc−/−*^ mice show patchy changes with a bronchiolocentric distribution characterized by peribronchiolar inflammatory infiltrates (black rectangles). (C) Alveolar parenchyma from a smoking WT mouse showing areas of emphysema (*) with a patchy distribution. (D) is higher magnifications of (B) showing in peribronchiolar areas a large number of macrophages within the adjacent alveoli. H&E stain. Scale bars = 200 μm.

The lungs of air-control *p66* ^*Shc−/−*^ mice exposed to air displayed a well fixed normal parenchyma with normal airways a normal architecture (Fig. 3A). After 7 months of CS exposure, the lungs of *p66* ^*Shc−/−*^ mice showed, trivial patchy areas of air space enlargement (Fig. 3B). Lm and ISA measurements, as well as biochemical analysis of lung elastin content (determined as desmosine) revealed no significant difference between air-exposed and smoking *p66* ^*Shc−/−*^ mice (Lm: 37.25 ± 0.68 *vs* 37.36 ± 0.63 μm, NS; ISA: 1,193 ± 50 *vs* 1,175 ± 57 cm^2^, NS; desmosine 2.92 ± 0.08 *vs* 2.89 ± 0.12 μg/lung, NS; respectively).

On the other hands, significant areas of patchy emphysema were prominent in the lungs of smoking 129/SVEv WT mice (Fig. 3C). In these mice, chronic CS exposure resulted in a significant increase of the Lm and a decrease of the ISA and lung desmosine content in respect to mice of the air-exposed control group (Lm: 45.35 ± 0.73 *vs* 37.68 ± 0.57 μm, p<0.05; ISA: 1,013 ± 57 *vs* 1,163 ± 52 cm^2^, p<0.05; desmosine 2.43 ± 0.11 *vs* 2.94 ± 0.07 μg/lung, p<0.05; respectively).

Although emphysematous changes in smoking WT mice appears of elastinolytic nature, no differences were found between WT and *p66* ^*Shc−/−*^ mice in the expression of some MMPs (i.e. MMP-1, MMP-9 and MMP-12) at 5 and 7 months of CS exposure (data not shown).

### Knock out of *p66* ^*Shc*^ gene in mice resulted in respiratory bronchiolitis with fibrosis after chronic exposure to CS

Of interest, the exposure to CS for 7 months in mice with the targeted mutation for *p66* ^*Shc*^ resulted in the development of mild inflammatory lung changes centering on respiratory bronchioles and neighboring alveoli (peribronchiolar air spaces) with sparing of more distal air spaces. In particular, these lesions showed a patchy distribution and consisted of peribronchiolar lymphocytic infiltrates and a large number of macrophages within the adjacent alveoli (Fig. 3B and D). Some of these lesions, even if to a lesser extent, could be appreciated in 4 out 8 smoking *p66* ^*Shc−/−*^ mice at 5 months after CS (data not shown). None of these changes were present in air control *p66* ^*Shc−/−*^ mice (Fig. 3A) and in mice at 3 months after CS.

**Fig 4:**
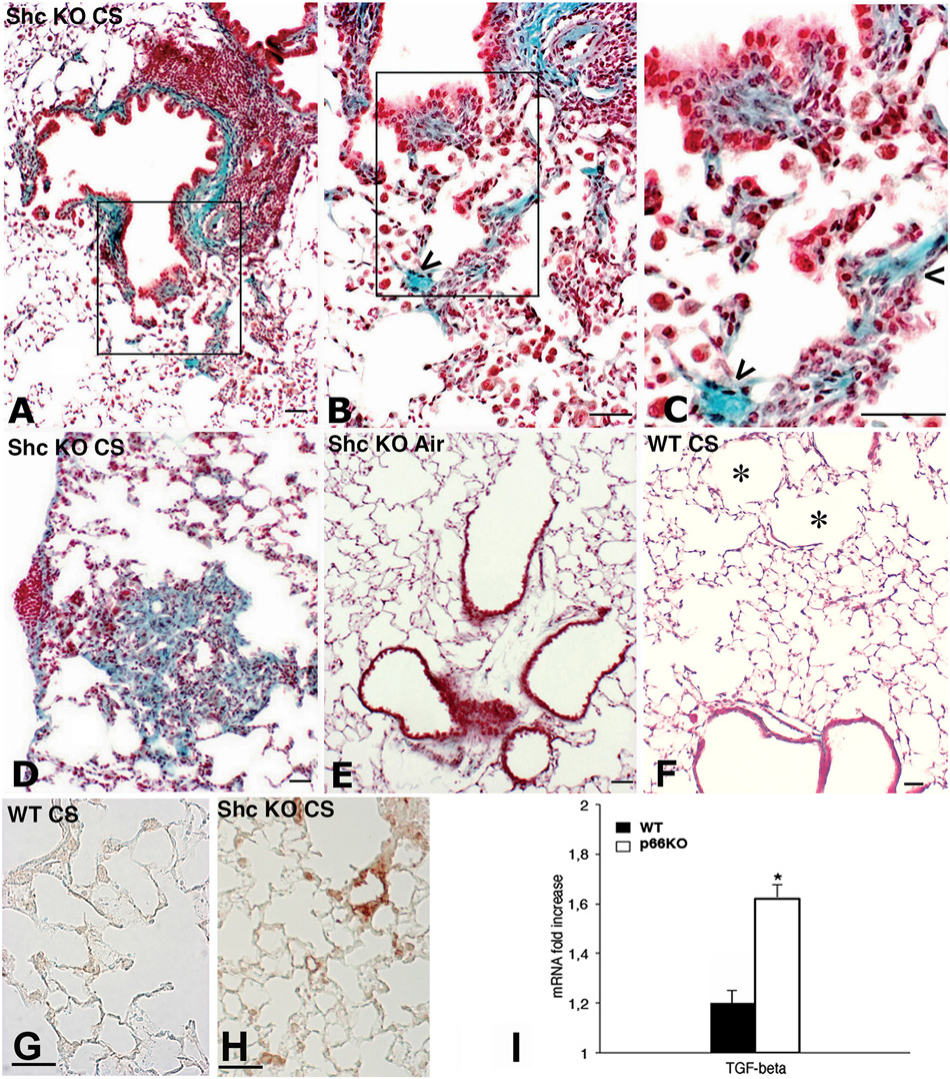
Representative lung micrographs and TGF- mRNA expression of p66Shc−/− mice and WT mice exposed to CS for 7 months. (A) and (D) are lung micrographs of a p66Shc−/− mouse at 7 months after CS exposure. (B) and (D) show morphologic appearance of peribronchiolar fibrosis, characterized by a progressive deposition of collagen expanding into contiguous alveolar septa. (B) and (C) represent higher magnifications of (A). These micrographs show intraseptal collagen accumulation (“sea green”) (arrowheads) and alveolar lumens, which are lined by hyperplastic epithelial cells and filled with macrophages. These macrophages are often multinucleated. (E) is representative lung parenchyma from an air-control p66Shc−/− mouse showing a normal appearance. (F) is a lung parenchyma from a WT mouse exposed to CS for 7 months, showing air space enlargements (emphysema) (*). Masson’s trichrome stain. Scale bars = 40 μm. Immunohistochemical staining for transforming growth factor-beta (TGF-) on lung sections of WT (G) and p66Shc−/− (H) mice at 7 months. (I) Real-time PCR analysis of mRNA for TGF-β carried out on lungs from six mice for each experimental group at 7 month after CS exposure. Values are corrected for 18S rRNA and normalised to a median control value of 1.0. Data are presented as meanSD. * P< 0.05 with air-control values of the same genotype.

At 7 months after CS, smoking *p66* ^*Shc−/−*^ mice developed peribronchiolar fibrosis (Fig. 4A and D), characterized by a progressive deposition of collagen, that expanded to contiguous alveolar septa (Fig. 4B and C). Alveolar lumens were frequently lined by hyperplastic epithelial cells, and were filled with macrophages, many of which were multinucleated (Fig. 4C).

This histological pattern was not observed either in air-exposed *p66* ^*Shc−/−*^ mice (4 E) or in WT mice exposed to CS or air for 7 months (Fig. 4F).

The increase of collagen deposition seen in smoking *p66* ^*Shc−/−*^ mice was accompanied by a marked increase of mRNA expression of TGF-beta in lung tissue (Fig. 4I) and a positive reaction for TGF-beta after immunohistochemical staining (Fig. 4H). This feature was not observed in WT mice (Fig. 4G and I). A slight but significant increase of hydroxyproline content was observed between smoking and air-exposed *p66* ^*Shc−/−*^ mice (161.91 ± 7.45 μg/lung *vs* 150.21± 6.65 μg/lung; p < 0.05). No difference in lung hydroxyproline content was found between air- and CS-exposed WT mice.

### Alveolar macrophages from smoking *p66* ^*Shc−/−*^ mice showed a M2 pattern of polarization

**Fig 5:**
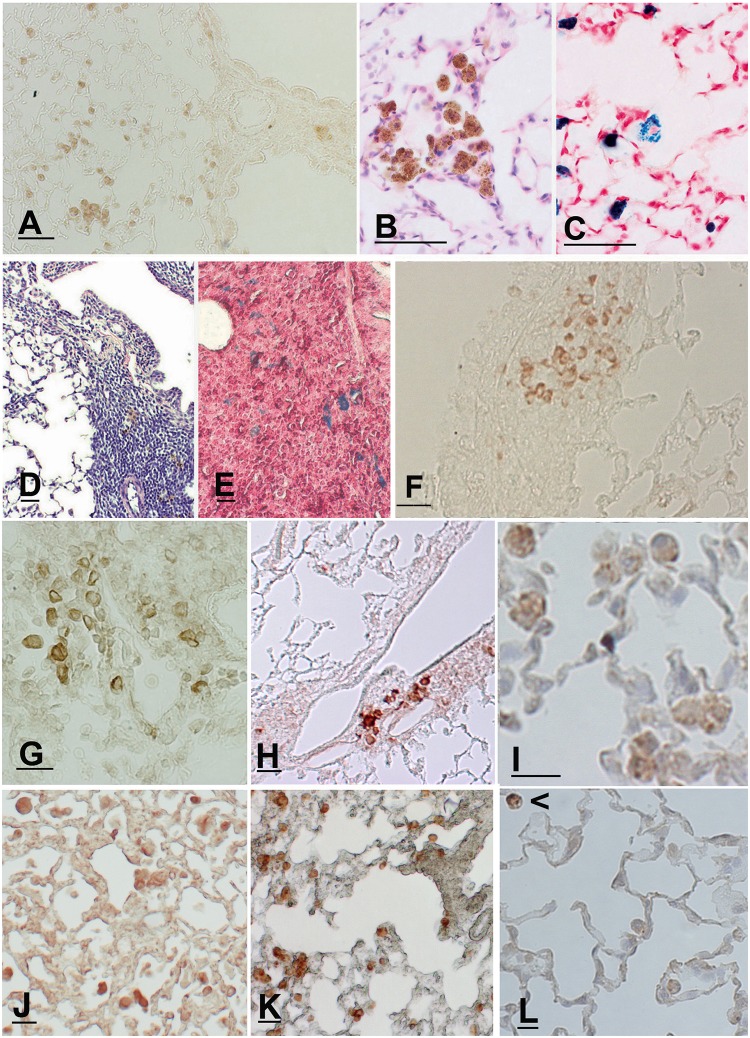
Histological lung sections of a *p66* ^*Shc−/−*^ mouse at 7 months after CS exposure. (A) Positive immunostaining for MAC-3 confirms that the free alveolar cells are predominantly macrophages. A small number of macrophages are also present in the peribronchiolar infiltrates. (B) Representative lung section showing alveolar multinucleated macrophages containing fine granular brown cytoplasmic particles that stain with Perls’ Prussian blue (C). (D) The peribronchiolar areas of inflammation are characterized by a large amount of lymphocytes and a small number of pigmented cells that also stain with Perl’s Prussian blue (E). (F) Many fascin positive cells (histiocytes) are present in the peribronchiolar areas. (A) and (F): Scale bars = 80 μm; (B-E): Scale bars = 40 μm. Immunohistochemistry of CD4 (G), IL-4 (H), IL-13 (I), Arginase I (J), Chitinase (K) and iNOS (L) in the lung tissue of *p66* ^*Shc−/−*^ mice at 7 months after CS exposure. In Fig. 4L, a M1 macrophage (iNOS positive)(arrowhead) is present together iNOS negative macrophages. (G-L): Scale bars = 40 μm.

The immunohistochemistry for MAC 3 antigen confirmed that the free alveolar cells observed in *p66* ^*Shc−/−*^ mice after CS exposure were predominantly macrophages (Fig. 5A), whereas in the peribronchiolar infiltrates we detected only a small number of macrophages (Fig. 5A) and many fascin positive histiocytes (Fig. 5F). The presence of histiocytes in these areas was also confirmed by the presence of S-100 positive cells (data not shown).

**Fig 6:**
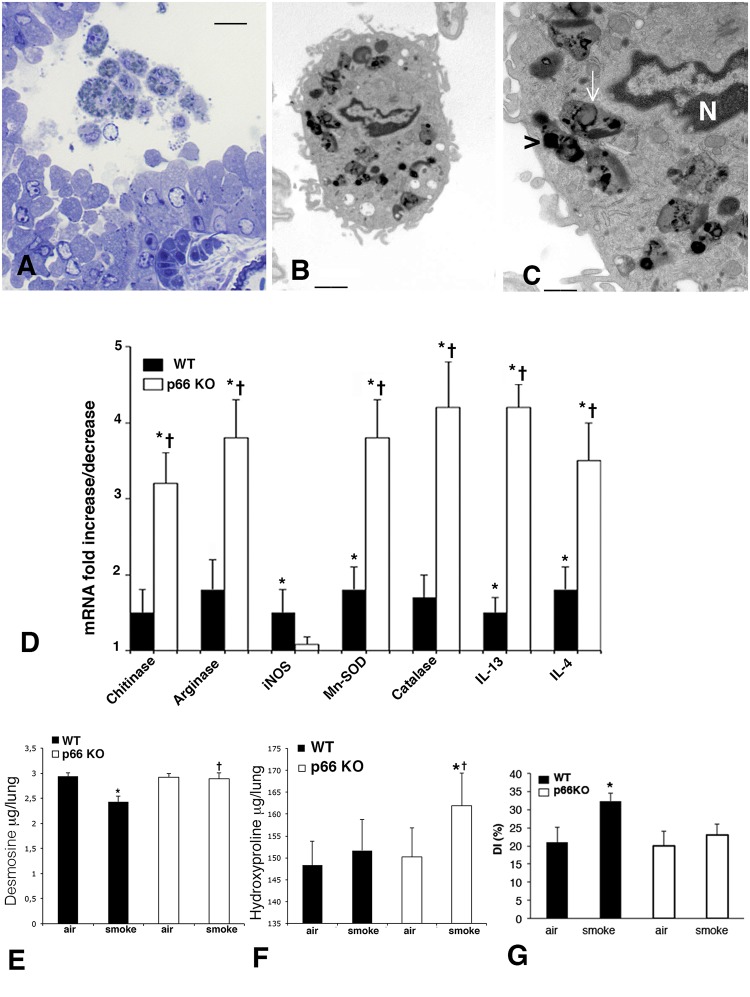
Macrophages TEM examination, Destructive Index, Lung Desmosine, and Hydroxyproline contents in smoking p66Shc KO. (A) Semithin section from a lung of p66Shc KO at 7 months after CS exposure showing in bronchiolar lumen several macrophages containing cytoplasmic inclusion. Toluidine blue Scale bar = 12 μm. (B, C) Lung macrophages from smoking p66Shc KO mice show at TEM examination an irregular outline and are loaded of single-membrane-bound bodies containing electron dense particles (black arrowhead) and lipofuscin granules containing small lipid droplets (white arrowhead). Uranyl acetate-lead citrate (B) Scale bar = 2 μm; (C) scale bar = = 0.8 μm. (D) Real-time PCR analysis of mRNAs for chitinase, arginase, iNOS, Mn-SOD, catalase, IL-4 and IL-13 carried out on lungs from 6 mice for each experimental group at 7 months (or 5 months, for catalase and Mn-SOD) after CS exposure. Values are corrected for 18S rRNA and normalized to a median control value of 1.0. Error bars indicate mean ± SD. *p <0.05 compared with air-control values of the same genotype; ^**†**^p< 0.05 *vs* smoking WT mice. (E) Lung Desmosine and (F) Lung Hydroxyproline expressed as μg/lung. Values are represented as means ± SD, *p < 0.05 compared with air-control values of the same genotype. ^†^p< 0.05 *vs* smoking WT mice. (G) Unlike *p66* ^*Shc−/−*^ mice, WT mice show a significant increase of destructive index (DI) (+11%) at 5 months after CS exposure.

The macrophages present in *p66* ^*Shc−/−*^ mice after CS exposure were multinucleated and contained fine granular brown cytoplasmic particles (Fig. 5B) that stained with Perls’ Prussian blue (Fig. 5C). In peribronchiolar inflammatory infiltrates, a small number of pigmented cells (Fig. 5D), which also stained with Perls’ Prussian blue (Fig. 5E), were present within a large number of lymphocytes. Many of peribronchiolar lymphocytes were positive for the surface antigen CD4 (Fig. 5G) suggesting for these cells a T helper function. The presence of Th2 cytokines such IL-4 (Fig. 5H) and IL-13 (Fig. 5I) in cells dispersed around lung vessels and in alveolar lung structures together with M2 macrophages expressing arginase I (Fig. 5J) and chitinase (Fig. 5K) suggest a predominant Th2 pattern of cell activation in *p66* ^*Shc−/−*^ lungs in response to chronic CS exposure. Only few macrophages interspersed within lung parenchyma showed a Th1 pattern of activation, as revealed by iNOS immunohistochemical staining (Fig. 5L). A marked increase in the expression of chitinase and arginase I as well as of IL-4 and IL-13 in lungs of smoking *p66* ^*Shc−/−*^ mice was found at 7 months after CS exposure (Fig. 6D). The expression of iNOS in lung of smoking *p66* ^*Shc−/−*^ mice was not significantly different form air control *p66* ^*Shc−/−*^ mice (Fig. 6D). Pigmented macrophages in smoking *p66* ^*Shc−/−*^ mice were present within (Fig. 6A) and around bronchioles. At TEM examination, lung macrophages containing inclusions were endowed with an active membrane, as evinced by the presence of fine pseudopodia and an irregular outline (Fig. 6B). They were also loaded of lysosomal bodies such as siderosomes and lipofucsin granules, Siderosomes are characterised by a single-membrane–bound bodies containing aggregates of electron dense hemosiderin particles (Fig. 6B and C), which probably correspond to Prussian-blue positive granules observed at light microscopy (Fig. 5C). Lipofuscin granules were composed of numerous electron dense granules containing small lipid droplets, limited by a single membrane (Fig. 6B and C).

These features were generally not observed in the cytoplasm of macrophages from smoking WT mice (data not shown). Desmosine and hydroxyproline lung values detected in the various groups of mice are reported in Fig. 6E and F. The value of DI are summarized in Fig. 6G.

### Lung macrophages and epithelial cells from smoking *p66* ^*Shc−/−*^ mice showed a reduced levels of apoptosis and oxidative DNA damage after CS exposure

**Fig 7:**
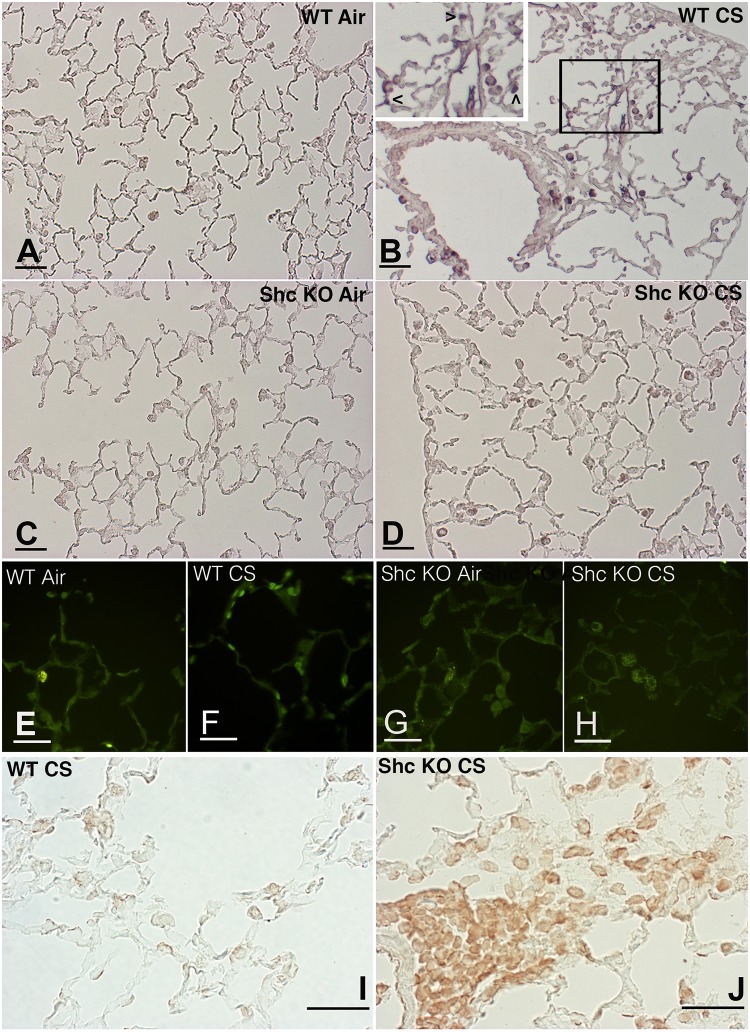
Lung slides from WT and *p66* ^*Shc−/−*^ mice scored at 5 months for active caspase-3, TUNEL and p16INK4a. Immunohistochemical evaluation of active caspase-3 on lungs of air control (A) and smoking WT (B) mice at 5 months. Note in (B) a positive staining on alveolar macrophages and epithelial lung cells. The positivity on lung epithelial cells is more evident at higher magnification (see the arrowheads in the inset). Active caspase-3 staining on tissue slides from air-control (C) and smoking *p66* ^*Shc−/−*^ mice (D). DNA strand-break extremities labeling (TUNEL) on lung tissues from air-control (E) and smoking (F) WT at 5 months. In (G) and (H) TUNEL labeling on air-control and smoking *p66* ^*Shc−/−*^ mice, respectively. Immunohistochemical staining for the cyclin-dependent kinase inhibitor p16INK4a on lung slides from WT (I) and *p66* ^*Shc−/−*^(J) mice after 5 months of CS exposure. (A-J): Scale bars = 40 μm.

**Fig 8:**
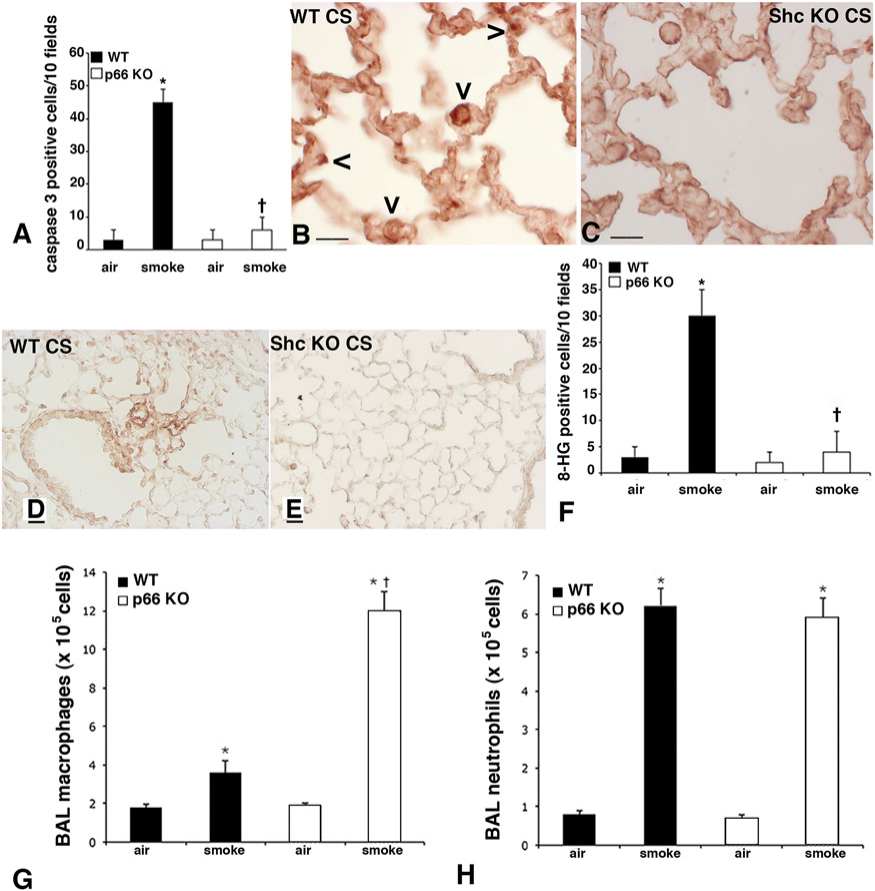
Immunohistochemical analysis of caspase 3-, ceramide-, and 8-HG- positive cells, as well as BAL cell counts in smoking mice. Quantification of caspase 3- positive cells in lungs after 5 months of CS exposure. Increased number of caspase 3- positive cells was detected in the lungs of CS-exposed WT mice. Few 8-HG-positive cells were detected in the lung tissues of air-exposed mice from both groups and in smoking *p66* ^*Shc−/−*^ mice. Values [positive cells/10 fields] are represented as means ± SD, *p < 0.05 compared with air-control values of the same genotype. ^**†**^p< 0.05 *vs* smoking WT mice. (B-C) Immunohistochemistry for ceramide in WT (B) and p66Shc KO mice (C) at 5 months after CS exposure. A positive reaction is evident on macrophages and alveolar epithelial cells in lungs from WT mice (arrowheads). Scale bars = 25 μm. (D-E) Immunohistochemistry for 8-hydroxyguanosine (8-HG). A strong 8-HG staining is present on lung tissue of WT mice at 3 months after CS exposure (D). A faint 8-HG reaction is found on lung slides from *p66* ^*Shc−/−*^ smoking mice at the same time point (E). (D, E): Scale bars = 40 μm. (F) Quantification of 8-HG-positive cells in lungs after 3 months of CS exposure. Increased number of 8-HG-positive cells was detected in the lungs of CS-exposed WT mice. Few 8-HG-positive cells were detected in the lung tissues of air-exposed mice from both groups and in smoking *p66* ^*Shc−/−*^ mice. Values [positive cells/10 fields] are represented as means ± SD, *p < 0.05 compared with air-control values of the same genotype. ^**†**^p< 0.05 *vs* smoking WT mice. (G-H) Macrophage (G) and Neutrophil (H) counts in bronchoalveolar lavage [BAL] fluids of mice immediately sacrificed after smoking at 7months of treatment. Values reported in are means ± SD. *p< 0.05 *vs* air controls of the same genotype; n = 5 animal/group. ^†^p< 0.05 *vs* smoking WT mice.

The exposure to CS in WT mice was associated by a significant increase of alveolar macrophage and lung epithelial cell apoptosis as revealed by a positivity staining for active Caspase-3 (Fig. 7B, Fig. 8A). This figure was evident in WT mice from 5 months onwards (Fig. 7B, inset), but it was not present in air-exposed WT mice (Fig. 7A) and in *p66* ^*Shc−/−*^ mice at various times after CS exposure (Fig. 7C, D and Fig. 8A).

At 5 and 7 months after CS exposure DNA fragmentation was evident after TUNEL staining in lung epithelial cells from WT mice (Fig. 7E and F) but not in those from *p66* ^*Shc−/−*^ mice (Fig. 7G and H). From 5 months onwards macrophages and alveolar epithelial cells of *p66* ^*Shc−/−*^ mice showed following smoking exposure high expression of p16INK4a (Fig. 7J). A faint reaction for this factor was found in parenchymal cells from WT at different time points after CS exposure (Fig. 7I).

Unlike smoking *p66* ^*Shc−/−*^ mice (Fig. 8E), smoking WT mice showed from 3 months onwards elevated levels of 8-HG, a marker for oxidative DNA damage (Fig. 8D). Quantitative evaluation revealed a significant (p < 0.01) increase in the number of 8-HG-positive cells in the lung sections of CS exposed WT mice in respect to those of *p66* ^*Shc−/−*^ mice (Fig. 8F). Immunohistochemical reaction for ceramide revealed a strong positivity in macrophages and alveolar epithelial cells in smoking WT mice at 5 (Fig. 8B) and 7 months after CS. No substantial positivity for ceramide was observed in lung slides from smoking p66Shc KO mice at the same time points (Fig. 8C). At 7 months, Mn-superoxide dismutase and catalase were highly expressed in lungs of smoking *p66* ^*Shc−/−*^ mice (Fig. 6D). These data offer an explanation for the differences we observed between the various strains of mice in terms of Destructive Index (DI) (Fig. 6G) as well as Lm and ISA values.

Although significant differences were observed in terms of oxidative damage (as revealed by 8-HG expression), DNA fragmentation (TUNEL positivity) and apoptosis (active caspase 3 positivity) under chronic conditions, no substantial changes in terms of oxidants/antioxidants balance (i.e. thiol status and TEAC) has been detected in BAL fluids between WT and *p66* ^*Shc−/−*^ mice after chronic CS (data not shown). After 7 months of CS exposure, a significant increase of the number of BAL macrophages has been observed in WT and *p66* ^*Shc−/−*^ mice. In the latter group a 6-fold increase of the macrophage number was seen (Fig. 8G). On the other hand, no difference between the two strains was found in the number of neutrophils at this time (Fig. 8H).

None of the WT and *p66* ^*Shc−/−*^ mice chronically exposed to CS died before the intended end of the experiment.

## Discussion

The data of this study suggest P66Shc ablation in mouse genome affords protection towards CS induced pulmonary emphysema. However, *p66* ^*Shc−/−*^ mice develop some pathological changes which have been reported to characterise in man Respiratory Bronchiolitis-associated Interstitial Lung Disease (RB-ILD)[37–39].

As mentioned above *p66* ^*Shc−/−*^ mice are resistant to oxidative stress [12, 15, 18–23] and show reduced levels of p53-dependent apoptosis [15]. It is well recognized that intracellular ROS alter the mitochondrial membrane potentials, leading to activation of mitochondrial permeability transition pore and release of cytochrome C. P66Shc regulates mitochondrial permeability, and hence cytochrome C release, by modulating production of ROS [40]. It is conceivable that reduced levels of apoptosis and the oxidative stress resistance under our experimental smoking conditions confer protection to *p66* ^*Shc−/−*^ mice against the development of emphysema, but result in unexpected pathology characterized by respiratory bronchiolitis with fibrosis. The findings reported in this paper suggest that the resistance to oxidative stress due to marked expression of antioxidant enzymes (such as catalase and Mn-SOD) and the blockage of apoptosis interfere with the macrophage “clearance” from alveolar spaces [41, 42]. This may favour the accumulation of aging macrophages into alveolar spaces and the progressive accumulation of iron pigment in long-lived senescent macrophages. This opinion is supported by high expression of p16INK4a in alveolar macrophages of *p66* ^*Shc−/−*^ mice. It is well recognized that cyclin-dependent kinase inhibitor p16INK4a is highly expressed during cellular senescence [43].

**Fig 9:**
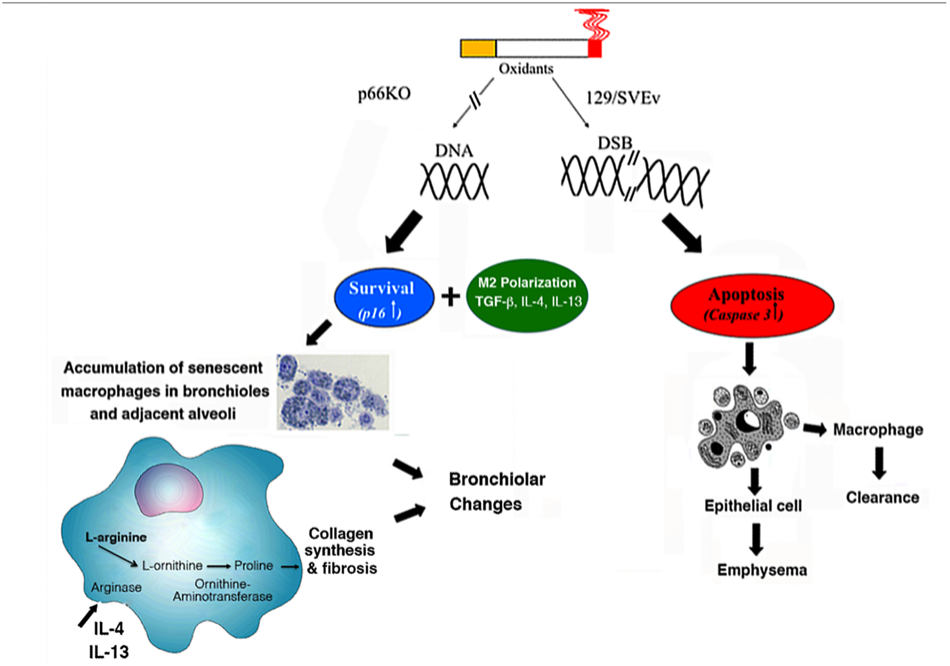
Hypothetical schematic model showing the switch from emphysema to bronchiolitis involving p66Shc and M2 macrophages. In WT mice, CS exposure causes DNA fragmentation and apoptosis of alveolar epithelial cells and macrophages leading to emphysematous changes and macrophage clearance. *p66* ^*Shc−/−*^ mice are protected from oxidative damage by increasing antioxidant enzymes and up-regulate the expression of p16INK4a, TGF-beta, IL-4 and IL-13, which promote accumulation of senescent macrophages and M2 polarization. The expression of Arginase I in M2 macrophages favours collagen synthesis and deposition in peribronchiolar areas and alveolar interstitium.

Alveolar macrophages in smoking *p66* ^*Shc−/−*^ mice show alternative pattern of activation (M2 phenotype) as revealed by Mac-3 as well as chitinase and arginase I hyperexpression [44, 45]. This may offer an explanation to the development of a pathological picture of respiratory bronchiolitis with fibrosis. A hypothetical schematic model showing the switch from emphysema to bronchiolitis involving p66Shc and M2 macrophages is reported in Fig. 9.

It has been recently reported that macrophages in COPD are polarized toward the proinflammatory M1 phenotype [46] and that monocytes from COPD patients may more prone to differentiate into M1 macrophages upon exposure to GM-CSF [47]. However, more recent studies carried out in patients provided partially conflicting results suggesting that macrophages in COPD constitute a heterogeneous population with an M1/M2 intermediate phenotype [47].

In mouse models, however, alveolar macrophages elaborate M1 signature cytokines in response to CS exposure in vivo, for review see: [48]. Unlike macrophages of other strains of mice, macrophages of *p66* ^*Shc−/−*^ mice elaborate under smoke exposure M2 cytokines (i.e. IL-4 and IL-13) and some enzymes such as chitinase and arginase I, which may favour collagen deposition and then fibrosis in the surrounding areas. Thus, a prevailing Th2 pattern dominates the immune response in smoking *p66* ^*Shc−/−*^ mice. This is confirmed by the presence of cell positive for IL-4 and IL-13. These factors may be involved in the pathogenic mechanism(s) leading to collagen deposition by influencing the expression of TGF-beta and the macrophage M2 pattern of polarization with hyper-expression of arginase I. TGF-beta1 by itself inhibits inducible NO synthase, stimulates arginase I and plays an important role in regulating the balance between M1 and M2 macrophages [49].

It is well recognized that classically activated macrophages (M1) express inducible nitric oxide synthase (iNOS), an enzyme that allows them to metabolize arginine into NO for microbial killing. In alternatively activated macrophages (M2), IL-4 induces arginase activity, which converts arginine to ornithine, a precursor of polyamines and proline, necessary for collagen synthesis [50]. This allows these cells to participate in wound healing and scarring. Additionally, hyperexpression of TGF-beta a central mediator in fibrosis promotes fibroblast recruitment and proliferation [51] as well as epithelial to mesenchymal transition [52]. Thus, M2 macrophages in smoking *p66* ^*Shc−/−*^ mice have a low destructive potential, produce lower amounts of proinflammatory cytokines and contribute to the synthesis of matrix components that leads to fibrosis (Fig. 9).

Although, we observed no substantial differences between WT and *p66* ^*Shc−/−*^ mice in terms of BALF inflammatory cells, total antioxidant capacity, oxidized glutathione, protein thiols and KC concentration after acute exposure to CS, we detect marked differences in lung responses following chronic CS between the strains of mice. In particular, WT mice develop anatomical emphysema in which the apoptosis of alveolar epithelial cells induced by the oxidative damage to DNA may play an important pathogenic role. The results of TUNEL test, caspase 3 and 8-HG staining we observed in alveolar epithelial cells of WT mice chronically exposed to CS strongly support this view. The elevated expression of ceramide in the lungs of smoking WT mice in respect to those seen in p66Shc KO mice may induce reactive oxygen species by altering mitochondrial function or indirectly by inactivating antioxidant enzymes such as catalase. It is therefore conceivable that elevated ceramide levels might trigger both alveolar epithelial and macrophage apotosis and expression of oxidative stress biomarkers such as 8-HG [9].

Unlike WT mice, *p66* ^*Shc−/−*^ mice do not develop emphysema and show a marked protection toward DNA damage induced by oxidants as revealed by trivial 8-HG staining on alveolar epithelial cells. This may explain the presence of positivity for caspase-3 and TUNEL test in very few alveolar epithelial cells of *p66* ^*Shc−/−*^ mice.

As mentioned above, *p66* ^*Shc−/−*^ mice after chronic exposure to CS mimic some histopathological traits, which have been recently associated in man with a smoking-related lung disease labelled “*Respiratory Bronchiolitis with fibrosis*” [37].

ROS initially considered as solely destructive agents have been recently shown to be involved in several specific signaling functions important for cell homeostasis. The block of these signals, as reported in our study, may result in smoking animals in unexpected pathology characterized by patchy lung changes with bronchiolocentric distribution with yellow-brown-pigmented macrophages within bronchiolar lumina, peribronchiolar inflammation and mild interstitial fibrosis. The “the dark side of antioxidants” is now emerging in the literature and a more recent study “suggests that high doses” of antioxidant supplements “may do more harm than good in patients with certain types of cancer.” [53].
